# Infantile Scurvy1Read at the meeting of the Elmira Academy of Medicine, September 5, 1906.

**Published:** 1907-02

**Authors:** C. W. M. Brown

**Affiliations:** Elmira, N. Y.


### BUFFALO MEDICAL JOURNAL.

VOL. LXII. FEBRUARY 1907. No. 7

### ORIGINAL COMMUNICATIONS.

INDEX THROUGH

### Infantile Scurvy.[1]

BY C. W. M. BROWN, M. D., Elmira, N. Y.

INFANTILE SCURVY is a disease of modern times and is attributable to altered conditions arising from over-civilization and from the crowding into cities, which makes it difficult or impossible for children to be fed in an ideal manner. There has been a steady diminution, during the last half century, in the number of children who are fed in the natural mammalian fashion,—at the mother's breast. It is less than thirty years since the nature of the disease was recognised by Cheadle, his first paper appearing in 1878. His article, in which he insisted on the identity of infantile and adult scurvy, was published in 1882, but his views were somewhat slow in gaining general acceptance, and the truth of his contention was not thoroughly established until a paper was read by Sir Thomas Barlow on this subject before the Royal Medical Society in 1883.

"Scurvy is a constitutional disease due to some prolonged error in diet." Among its symptoms are spongy, bleeding gums, swelling and ecchymoses about the joints, especially the knee and ankle, hemorrhage from the nose and occasionally from other mucous membranes, hematuria, extreme hyperesthesia and often pseudoparalysis of the lower extremities. These local symptoms are usually accompanied with anemia. Scurvy and rickets are frequently associated, but are not necessarily connected and are not different forms of the same disease. A large majority of the cases occur between the sixth and fifteenth months and half of them between the seventh and tenth months. The greater number are seen in private practice, often in the midst of the best surroundings. The only important etiological factor yet known to bear any relation to scurvy is the diet. The most marked effects of scurvy are seen in the bones, bloodvessels and the blood. The most marked lesion is sub-periosteal hemorrhage and may occur almost anywhere in the body, but chiefly affects the bones of the

1. Read at the meeting of the Elmira Academy of Medicine, September 5, 1906.

lower extremities and may reach from the knee to the great trochanter, or from the ankle nearly to the knee. Effusions may also be found between the muscles and blood may infiltrate the cellular tissue in the neighborhood of the joints. The bones themselves may also be affected. Separation of the epiphyses of some of the long bones, generally at the lower end of the femur or lower end of the tibia, is found in most of the fatal cases.

In cases, which have been carefully observed, there may be noticed before the evidences of scurvy appear a pallor, general indisposition and fretfulness, with failing nutrition, but usually tenderness of the bones is the first symptom noticed. At first it may be only slight and indefinite in character, so as to cause the infant to cry upon handling. Later, it becomes constant and more acute. Swelling may appear about the ankles and knees. There may be some changes in the gums, which are more apt to appear if the child has teeth. They are of a purplish color, swollen, bleed upon the slightest rubbing, sometimes spontaneously. Ulcerations are more apt to appear about the upper teeth. The child is cross, sleeps badly, loses color, weight, and appetite. Symptoms usually come on gradually and may continue for some weeks without any very marked impression upon the child's general condition. Not infrequently, however, symptoms appear rather suddenly, when they are usually ascribed to an injury, real or supposed. If the symptoms are not recognised, the pain and tenderness of the bones increase so that the child will lie motionless and no voluntary movement can be excited. Pain and tenderness are present in 95% of the cases. Paralysis is often suspected. Ecchymoses are frequently seen, especially about the large joints and often confirm the opinion previously formed, that the child has met with some accident. Bleeding may occur from the mouth, pharnyx, or bowels. Blood may be vomited or passed in the urine.

The urine was very carefully observed in 35 of Morse's cases. It was normal in 20 and abnormal in 15. In three of these, however, it was merely noted that it stained the diapers red or brown. Excepting pallor, it was the first symptom noted in eight of the proved cases of scurvy and was the only symptom in two. Unless recognised the symptoms increase in severity and conditions grow steadily worse until death occurs from general weakness, sudden heart failure, or from some complicating disease, such as bronchopneumonia or acute gastroenteritis. Separation of the epiphyses from the shaft of some of the long bones generally at the lower end of the femur or tibia is found in most fatal cases.

In 1898 the American Pediatric Society made a collection embracing 379 cases. Of these, twelve had breast milk, alone, in ten; five, raw cow's milk, without any other food in four; twenty, pasteurized milk—sixteen without any other food; sixty, condensed milk—thirty-two with no other food; one hundred and seven, sterilised milk—sixty-eight without any other food; and two hundred and fourteen had proprietary infant foods.

Cheadle gives details of the previous feeding in sixty cases of infantile scurvy observed by him; of these, forty-six were taking various patented foods, thirteen of these having a certain amount of milk said to be fresh, at the same time; and among the remainder three were taking peptonised and seven sterilised or humanised milk.

Morse, of Boston, in a recent article, reporting fifty cases occurring in his own practice, says: "From the point of view of modern ideas of reasonable infant feeding the food was irrational, in nineteen of the cases taking proprietary foods, in four of those taking milk mixtures and in the case of one on general diet, making a total of twenty-four. Milk was used in the preparation of the food in forty-one cases, while no milk was used in eight cases. In the forty-one cases in which milk was used in the preparation of the food, the mixture was boiled in twelve, pasteurised in nineteen and unheated in nine, while in one case there was no data as to whether or not heat was used. The food was also peptonised in four of the pasteurised and one of the unpasteurised mixtures. The mixture was too weak in five cases and the same mixture was continued for months in two cases. In only one case was a rational mixture being taken and that was prepared with barley water instead of water. The digestion was good in twenty-three and feeble in twenty-seven of these cases, apparently showing that the scurvy was not due to disturbance of digestion and that foods may cause scurvy without causing disturbance of digestion."

A short time ago there was an epidemic of infantile scurvy in Berlin, among children supplied with pasteurised milk from an institution, but it was found to be confined to children whose parents, as an additional precaution, boiled it for some time after it was delivered to the house. Out of twenty-five severe cases seen by Colman in hospitals and private practice, nineteen were taking some kind of dried infant food as staple diet, seven having a certain amount of fresh milk as well, the remaining six were taking sterilised milk or humanised milk, or milk sterilised by long heating.

The disease with which infantile scurvy is most frequently confounded, is rheumatism. Holt says that fully four-fifths of the cases which have come to his notice, have had that diagnosis made. The extreme rarity of rheumatism under one year should always make one cautious, and pain and tenderness in the legs only should in an infant invariably suggest scurvy rather than rheumatism. He says that many cases of scurvy come into the hands of the orthopedic surgeon. He has known a diagnosis of malignant disease to be made from the cachexia or discoloration and the pain. He has known two cases to be operated on by eminent surgeons, one with the diagnosis of sarcoma, and one of ostitis of both tibiæ.

The cases probably which are most easily missed are the slight cases where there is only hematuria or epistaxis present. Of the fifty cases reported by Morse, a correct diagnosis had been made in but five. The diagnosis in the other cases were acute nephritis 1, excess of uric acid with consequent staining 2, arsenical poisoning with inflammation of the kidneys 1, rickets 2, spinal or Potts disease 6, hip disease 1, periostitis 1, rheumatism 6, gout 1, syphilis of cord 1, difficult dentition 5—in four of which the gums had been lanced,—strain 1, injury 1, tuberculosis 1, gumma of eye 1. In two other cases the physicians stated they had no idea what was the trouble. While Morse himself states that he has mistaken congenital syphilis and hematuria from lead poisoning for scurvy.

Recently, Snow, of Buffalo, reported a very interesting case of hemorrhage into the orbit, with otherwise typical symptoms of scurvy, which had been seen by eight ophthalmologists without a correct diagnosis having been made. Yet the diagnosis of scurvy seldom presents any difficulty to one having once seen a case. If the essential features of the disease are kept in mind,—the soreness of the limbs, spongy, swollen gums, swelling near the large joints, a tendency to hemorrhage and a history of the prolonged use of some proprietary infant food, or sterilised, pasteurised or condensed milk. Scurvy is frequently associated with rickets. Of 340 cases reported in the American Pediatric Society investigations, in which this point was noted, symptoms of rickets are present in 152 or 45%, while in 55% rickets were absent. It is stated that in fifty of these cases the rickets antedated from the development of the scurvy. There were signs of rickets in forty-one cases out of fifty observed by Morse. The association of the two diseases is so frequent that by many of the English writers they are still spoken of as one disease—scurvy-rickets.

The prognosis is always good if the disease is recognised early. No patients with symptoms so serious improve with such marvelous rapidity as those with scurvy under prompt management. It is only when the disease is of long standing and the malnutrition is severe, or when serious complications involve the digestive tract, that the issue becomes doubtful. Any case allowed to go on may result fatally. It is rare that scurvy leaves any permanent effect. Recovery is not only rapid but complete and relapses are extremely rare.

The treatment is simple; stop all proprietary foods, condensed milk, sterilised milk, and substitute a diet of fresh cow's milk adapted to suit the child's digestion. With this treatment, improvement will soon begin and recovery will follow. However, the addition of fresh fruit juice, preferably orange juice, is of the greatest value and when given improvement is much more rapid. From half an ounce to four ounces may be given during the twenty-four hours. Other things of value are fresh beef juice, and for older children fresh vegetables and potatoes. The general condition, anemia, and malnutrition, should receive the proper attention. The fact remains then that when a child is artificially fed this can nearly always be satisfactorily obviated by supplying the child with milk as little altered as possible after it is drawn from the breast of the animal supplying it.

The diet statistics quoted show that while scurvy may develop occasionally upon almost any sort of food, several stand out more prominently—namely, pasteurised, sterilised, and condensed milks and proprietary infant foods. Analysis of these tables seem to show that the absence of freshness and the heating of the food are very important elements in the production of scurvy. The farther food is removed in character from the natural food of a child, the more likely is its use to be followed by the development of scurvy. Long continued use of the same percentages, at one time proper, has been followed by the appearance of scurvy. Formulæ should be changed from time to time as needed, and if patent foods and heated or condensed milks are used, their effects should be carefully watched, and they should not be continued as the sole diet for long periods. When scurvy and other diseases of nutrition have been proven to depend so completely upon diet, especially upon the use of patented foods, pasteurised, sterilised, or condensed milk, is it stating the fact too strongly to say that the use of these foods is radically wrong, may we not say it is almost criminal, for continuous infant feeding, when fresh, clean, cow's milk can be obtained?

In 1900 I reported to the Academy a fatal case of scurvy seen in consultation. The child was eleven months old and had the following history: was well since birth until about nine months of age, when a week or two after a supposed contusion the thigh began to swell and when seen was several times larger than the other. The skin was red, somewhat hotter than normal, and apparently fluctuated under palpation. The child was anemic, much emaciated and for several days had had enterocolitis. The gums were generally swollen and purple, especially upon the upper jaw. The child had ten teeth. The food from birth until a few days before I saw it had been sterilised milk and Mellin's Food. The attending physician had made a diagnosis of osteitis or periostitis with a large collection of pus. In this view I coincided. The next day the femur was opened under anesthesia and the periosteum was found separated from the whole length of the shaft, the shaft itself being separated from the epiphysis. The separation of the periosteum from the shaft was made by effused blood, which was also found extravasated between the muscles. The child was very weak and died two days later from exhaustion. This case, as will be seen, presented a typical picture of scurvy.

A few months after that I saw at the New York Polyclinic a similar case, where the tibia alone was affected. This had been opened by a surgeon connected with the Babies' Hospital, who saw it before a correct diagnosis had been made. A wonderfully rapid improvement was made in this case by the use of orange juice, beef juice and fresh milk. The leg healed rapidly under antiseptic dressing. In this case, as in the one seen in consultation, the child had been fed on sterilised milk and Mellin's Food.

Case No. 3, a child somewhat over two years of age, was fed the first year on Mellin's Food, with the history of having done well for nine months, and later upon malted milk and finally upon Eskay's Food, when I first saw it at the age of about eighteen months. The digestion was exceedingly poor and the changes suggested in the food were imperfectly or improperly carried out, because the mother repeatedly and persistently overfed the child, both in quantity and quality, so that at no time was it properly and wisely nourished. During one of its temporary more acute attacks of indigestion the gums became purple, tender, swollen, and bled easily. No other symptoms were manifested. The administration of orange juice promptly relieved it.

Case No. 4 was seen in May, 1903. The following history will be drawn from a private letter recently received from the mother: "Was nursed for four weeks, but did not gain an ounce. Was weaned on that account, because the milk seemed of such poor quality. She was then fed malted milk, with the addition of plain milk, until she was three months old, then as she had not gained at all on that, was given Mellin's Food and milk, as my other two children had been brought up on that and I thought I understood the management of it perfectly. That food was continued until the case was put into your hands. She was about nine months old when you saw her. The trouble, which caused your being called in, was the pain in her limbs whenever she moved them, which had begun about a week before, and that was the only symptom we had noticed, except that she had gained but a very few pounds since birth. She had had no previous illness, except occasional restlessness and fretfulness when she was cutting teeth. She had three upper and two lower teeth. I never saw anything work so like a charm as the orange juice you prescribed for her. In twenty-four hours the pain in her limbs was entirely gone." I may say in addition to the mother's story that the child was pale, flesh soft and flabby. Besides the orange juice she was given a properly modified cow's milk and improved both as to the scurvy and general nutrition as fast as could be anticipated, under the circumstances. I may add that the case had been treated for rheumatism by the attending physician.

Case No. 5, baby, age six months, born of healthy parents. Seen May 6th, 1906, when the following history was obtained: Nursed one week, fed condensed milk three weeks. At four weeks the baby was put upon diluted cow's milk. Mother cannot tell just how it was fixed. However, just before the baby was taken sick the mixture was "one-third milk, some sugar and the rest water." Besides, she says, the milk was dirty, poor in quality and kept badly. Baby often had colic, sometimes severe. Was given castoria every night, occasionally castor oil. April 26, the feet and legs began to swell and he had high fever and cried upon being lifted or being moved. I saw him May 6, when the feet and limbs were swollen half way from the ankles to the knees, and also both wrists. Cried lustily whenever feet or hands were touched. Abdomen distended with gas; temperature was 103°. Milk mixture was omitted, two teaspoons castor oil were given, no food until morning, then two teaspoons cream in four ounces water, with the juice of a whole orange in the twenty-four hours. Next ray, May 7, there was still much abdominal distension and pain. The stools contained a very large amount of mucus, in fact, were almost pure mucus. Temperature still 103, but the feet and limbs were much less swollen and painful. Because of severe enteritis all milk was omitted and diet was restricted to barley water. Castor oil was continued every day in teaspoonful doses. He was still more comfortable on May 8, when the orange juice was omitted because of the gastroenteritis, and he was given a tablespoon of raw beef juice three times a day, in addition to the barley water, the bowels were irrigated with normal saline solution twice a day. Because of the severe enteritis, improvement was gradual. The temperature did not become normal until May 16, when the swelling in the ankles was practically gone and by the 20th he was taking a milk mixture containing 2% fat, 1% proteid and 5½% sugar. Was free from colic and other evidences of indigestion and in one month had good color and had made a marked gain in weight. In this case the food contained the proper elements, but they were badly proportioned and perhaps had some toxic property because of its poor keeping quality. Two physicians in succession had been in attendance upon the case and both had called the disease rheumatism.

